# Acute kidney injury and COVID-19

**DOI:** 10.1186/s43162-021-00064-x

**Published:** 2021-10-06

**Authors:** Hayder M. Al-kuraishy, Ali I. Al-Gareeb

**Affiliations:** Department of Clinical Pharmacology, Medicine and Therapeutic, Medical Faculty, College of Medicine, Al-Mustansiriya University, P.O. Box 14132, Baghdad, Iraq

**Keywords:** COVID-19, SARS-CoV-2, Acute kidney injury

## Abstract

**Background:**

Coronavirus disease 2019 (COVID-19) is a recent pandemic infectious disease caused by severe acute respiratory syndrome coronavirus (SARS-CoV-2). COVID-19 may lead to acute kidney injury (AKI).

**Main text:**

SARS-CoV-2 uses angiotensin-converting enzyme 2 (ACE2) and dipeptidyl peptidase 4(DPP4) as entry point receptors in the alveolar type II cell of the lung. However, the expression of ACE2 is 100-fold higher in kidney tissue than the lung, though the potential entry point of SARS-CoV-2 for renal tissue and induction of AKI remains undefined. Therefore, reduction of ACE2 and high circulating angiotensin II in COVID-19 may together participate in the induction of AKI. Thereby, direct ACE2 activator is under investigation to be used as an effective therapy in the management COVID-19-induced AKI. Besides, the direct effect via invasion of SARS-CoV-2 may lead to glomerulopathy and renal proximal tubular necrosis.

**Conclusion:**

COVID-19 may associate with AKI due to direct effect of SARS-CoV-2 through ACE2 and DPP4 receptors or indirectly through the development of cytokine storm. Both ACE2 and DPP4 are interacted mutually in the pathogenesis of AKI. Thus, DPP4 inhibitors or ACE2 activators could reverse early AKI in COVID-19. Therefore, emerging of clinical trials is warranted to confirm the role of ACE2 and DPP4 modulators in COVID-19-induced AKI.

## Background

Acute kidney injury (AKI) is a rapid reduction in the renal functions due to renal ischemia, inflammatory reactions, and acute renal tubular injury that caused by pre-renal, renal, and post-renal causes [1]. Coronavirus disease 2019 (COVID-19) is a recent pandemic infectious disease caused by severe acute respiratory syndrome coronavirus (SARS-CoV-2), which started in Wuhan, China. COVID-19 was declared by the World Health Organization (WHO) as a pandemic disease on March 11, 2020. As of the middle of July 2021, there are more than 190 million confirmed cases with more than 4 million deaths worldwide [2]. Xiao et al. found that COVID-19 is associated with AKI, with an incidence of 0.5–80% mostly in old age patients with preexistence cardio-metabolic disorders. Lymphopenia and high D-dimer serum level with impaired liver function are at high risk for progress of AKI in patients with COVID-19 [3].

## Main text

Severe acute respiratory syndrome coronavirus (SARS-CoV-2) uses angiotensin-converting enzyme 2 (ACE2) as an entry point receptor in the alveolar type II cell of the lung. However, the expression of ACE2 is 100-fold higher in kidney tissue than the lung tissue [3], though the potential entry point of SARS-CoV-2 in the renal tissue and induction of AKI remains undefined. ACE2 is involved in the metabolism of angiotensin II (Ang II), which induces renal injury through triggering of inflammation and apoptosis [4]. ACE2 is a renoprotective receptor, and its downregulation by SARS-CoV-2 may lead to AKI through augmentation of intra-renal Ang II activity [5]. Consequently, decline of ACE2 with high circulating Ang II in COVID-19 may together contribute in the initiation of AKI. So, direct ACE2 activator is under investigation to be used as an effective therapy in the management COVID-19-induced AKI [6]. It has been shown that genetic variations in the expression of ACE2 may affect circulating Ang II and renal impairment, which might explain the higher COVID-19 severity in African-American population compared to Western population [6].

Taken together, Li et al. illustrated that patients with COVID-19 pneumonia showed proteinuria (63%), elevated serum creatinine (19%), and high blood urea nitrogen (27%). Also, the nucleic acids of SARS-CoV-2 are detected in the urine of patients with COVID-19 [7]. Besides, the computed tomography (CT) scan illustrated edema with significant inflammation of the renal parenchyma in all of the infected patients (100%) with SARS-CoV-2 infection [7]. These findings suggest the association of kidney tissue in COVID-19.

Contrariwise, Wang et al. demonstrated that the analysis of 116 patients with COVID-19 pneumonia from Wuhan, China, was not associated with AKI [8]. Amid large body of literatures that confirm the association between COVID-19 pneumonia and AKI, Cheng et al. showed that a sequential investigation of 710 patients with COVID-19 pneumonia at the Wuhan Educational Hospital clarified the existence of proteinuria (44%), hematuria (26.9%), and high blood urea nitrogen (14.1%) that were considered as independent risk factors for mortality in the hospitalized COVID-19 patients [9]. In recent times, AKI is more evident and affects about 50% of hospitalized COVID-19 patients, and often even recovered patients have imperfect renal function [10].

Indeed, the fundamental causes of AKI in COVID-19 pneumonia are multifactorial including dehydration, reduction of glomerular filtration rate (GFR), renal ischemia, hypoxia, rhabdomyolysis, hyper-inflammation, cardio-renal syndrome, and cytokine storm [11]. Further, endotheliitis, microangiopathy, and disseminated intravascular coagulation (DIC) that are induced by SARS-CoV-2 proteins lead to endothelial dysfunction and thrombosis which contribute to the pathogenesis of AKI [11].

Of interest, viremia and direct invasion of SARS-CoV-2 lead to glomerulopathy and renal proximal tubular necrosis with noteworthy renal endothelial injury [12]. Furthermore, uncontrolled use of analgesic drugs such as non-steroidal anti-inflammatory drugs (NSAIDs) in the management of COVID-19 patients may cause AKI through inhibition of vasodilator prostaglandin [13]. Besides, cardio-renal syndrome particularly right-side heart failure secondary to COVID-19 pneumonia might lead to renal congestion and AKI [14]. Yet, whether AKI induced by COVID-19 pneumonia is caused by SARS-CoV-2-induced cytopathic effect or by cytokine storm-induced glomerulopathy is still obscure [15]. Xu et al. study based on single-cell transcriptome analysis illustrated a robust evidence for the underlying mechanism of SARS-CoV-2-induced AKI through invasion of renal ACE2 [16]. Moreover, surface protein (SP) of SARS-CoV-2 is activated by trans-membrane serine proteases (TMPRSS2), which facilitate the binding of SARS-CoV-2 with ACE2 [17]. Both of ACE2 and TMPRSS are highly expressed in the proximal renal tubules and podocytes that increase their vulnerability to the toxic effect of SARS-CoV-2 [16]. Also, there are miscellaneous pathological findings in COVID-19-induced AKI including collapsing glomerulopathy, acute tubular injury, interstitial nephritis, and thrombotic microangiopathy due to direct SARS-CoV-2 invasion and/or cytokine storm [12].

It has been reported that the frequency of AKI in COVID-19 was higher in the western population than the Asian one due to a higher expression of ACE2 in the podocytes of Western populations [18]. Likewise, polymorphism and genetic variants for TMPRSS2 might be behind the severity of COVID-19 among Italian patients [19].

The plausible mechanisms of acute respiratory distress syndrome (ARDS) and AKI in COVID-19 might be related to the lung-kidney axis [20]. Ronco et al. observed that 68% of patients with ARDS develop AKI due to upregulation of IL-6 via injured renal tubular epithelium by which kidney and lung damages are developed [21]. In addition, high IL-6 is linked with alveolar damage and induction of capillary permeability with pulmonary hemorrhage [22]. Therefore, extraordinary circulating IL-6 is deemed to be the possible link AKI with ARDS in COVID-19 [21]. Hence, suppression of IL-6 may be a prime therapeutic regimen in COVID-19, as low molecular heparin is effective in management of COVID-19 through inhibition of IL-6 [23].

**Fig. 1:**
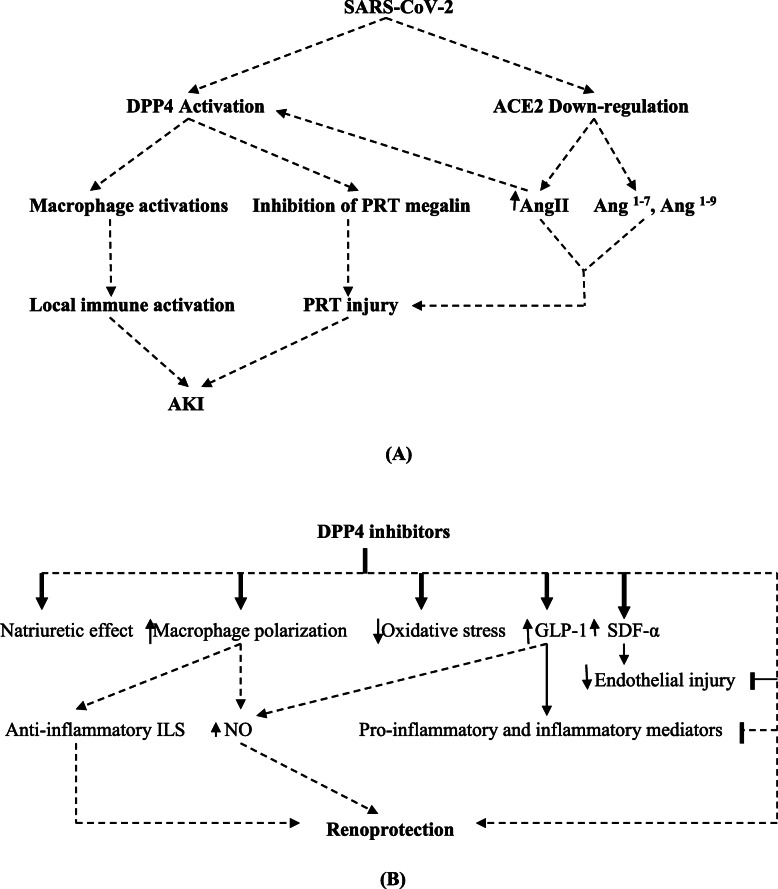
Acute kidney injury in COVID-19; role of ACE2, DPP4, and DPP4 inhibitors. **A** Role of ACE2 and DPP4 in the pathogenesis of AKI. **B**: Role of DPP4 inhibitors in renoprotection. GLP1, glucagon like peptid-1; SDF-α, stromal-derived factor alpha; NO, nitric oxide; PRT, proximal renal tubule; Ang, angiotensin; DPP4, dipeptidyl peptidase 4

To our acquaintance, a higher expression of kidney ACE2 is not utilitarian to explain and clarify AKI in COVID-19. Also, the systemic effect of cytokine storm is not merely affecting the kidney tissue. It has been reported that kidney proximal tubule cells express different serine proteases such as dipeptidyl peptidase 4(DPP4), cysteine protease cathepsin, and glutamyl-aminopeptidase that facilitate the entry of SARS-CoV-2 [24]. Also, DPP4 receptor is regarded as a potential entry point for SARS-CoV-2, thereby ACE2/DPP4 axis interacts meticulously in the pathogenesis of AKI [25]. Bardaweel et al. illustrated that DPP4 inhibitor sitagliptin attenuates COVID-19 and its complications like cytokine storm and AKI in diabetic patients with preexistence cardiovascular disorders [26]. Moreover, high circulating Ang II in patients with COVID-19 activates renal expression of DPP4 which is causing glomerular and tubular injury (Fig. 1A) [27]. Such effect is partly reversed by the administration of DPP4 inhibitors that modulate kidney inflammation and oxidative stress (Fig. 1B).

Of note, hypertensive COVID-19 patients that are commonly treated by ACEIs or angiotensin II receptor blockers (ARBs) are at higher risk of AKI. The increase in blood urea nitrogen with ACEI/ARB use could predict the progression of ARDS and respiratory failure [28]. A recent meta-analysis revealed that ACEI/ARB use is significantly linked with a higher incidence of AKI in hospitalized COVID-19 patients [29]. So, careful and vigilant monitoring of kidney complications is commended for COVID-19 patients with the recent use of ACEIs or ARBs due to the possible risk of AKI [30].

On the other hand, 20% of COVID-19 patients admitted to the intensive care unit need renal replacement therapy [31]. Therefore, diagnosis of AKI in COVID-19 and use of therapeutic and preventive measures to limit development of AKI are central to reduce morbidity and mortality in the lack of specific therapeutic options [31]. The management strategy for AKI in COVID-19 is mainly supportive by avoidance of nephrotoxic drugs with regular monitoring of urine output, blood urea, and serum creatinine as well as strict hemodynamic monitoring in critically ill COVID-19 patients [32]. In addition, application of lung-protective ventilation in COVID-19 patients reduces risk of AKI limiting ventilation-induced hemodynamic disturbances and cytokine burden on the kidney [33]. Indeed, low molecular weight heparin (LMWH) and appropriate antibiotics are necessary to prevent thrombosis and sepsis-mediated AKI respectively. Also, application of hemoperfusion might attenuate cytokine storm-induced AKI in critically ill patients with underlying immune dysregulation when other supportive treatments are insufficient or failing [34].

Taken together, there are limiting evidences concerning the potential link between AKI and COVID-19 as evident in most of the published studies (Table 1).

**Table 1:** The association between acute kidney injury (AKI) and COVID-19 pneumonia

References	Type of the study	Findings
Xiao et al. [3]	A single-center retrospective observational study	The old age, male gender, sepsis, lymphopenia increase risk of AKI in COVID-19
Li et al. [7]	Retrospective study	Patients with COVID-19 pneumonia showed proteinuria (63%), elevated serum creatinine (19%), and high blood urea nitrogen (27%)
Wang et al. [8]	Prospective study	AKI is uncommon in COVID-19 pneumonia
Cheng et al. [9]	Cohort study	Proteinuria, hematuria, and high blood urea nitrogen are considered as independent risk factors for mortality in the hospitalized COVID-19 patients
Sharma et al. [12]	Case series	COVID-19-induced AKI through collapsing glomerulopathy, acute tubular injury, interstitial nephritis, and thrombotic microangiopathy
Pan et al. [16]	Single-cell transcriptome analysis	SARS-CoV-2-induced AKI through invasion of renal ACE2
Asselta et al. [19]	Systematic review	The frequency of AKI in COVID-19 was higher in occidental population
Shi et al. [23]	A retrospective cohort study	Suppression of IL-6 may be a prime therapeutic regimen in COVID-19
Bardaweel et al. [26]	Systematic review	DPP4 inhibitor sitagliptin attenuates COVID-19-induced AKI
Valencia et al. [27]	Systematic review	Higher expression of DPP4 is linked with development of AKI
Oussalah et al. [28]	Retrospective longitudinal cohort study	COVID-19 patients chronically treated by ACEIs or angiotensin II receptor blockers (ARBs) are at higher risk of AKI
Lee et al. [29]	A meta-analysis study	ACEI/ARB use is significantly linked with a higher incidence of AKI in hospitalized COVID-19 patients

DPP4, dipeptidyl peptidase 4; ACEIs, angiotensin-converting enzyme inhibitors; ARBs, angiotensin II receptor blockers

## Conclusions

COVID-19 may be associated with AKI due to direct effect of SARS-CoV-2 through ACE2 and DPP4 receptors or indirectly through the development of cytokine storm. Both ACE2 and DPP4 are interacted mutually in the pathogenesis of AKI. Thus, DPP4 inhibitors or ACE2 activators could reverse early AKI in COVID-19. Therefore, emerging of clinical trials is warranted to confirm the role of ACE2 and DPP4 modulators in COVID-19 AKI.

## Data Availability

Nil

## Abbreviations

COVID-19: Coronavirus disease 2019
AKI: Acute kidney injury
SARS-CoV-2: Severe acute respiratory syndrome coronavirus
ACE2: Angiotensin converting enzyme 2
